# Predictors of Self-rated Health and Lifestyle Behaviours in Swedish University Students

**DOI:** 10.5539/gjhs.v4n4p1

**Published:** 2012-05-15

**Authors:** Manuela Schmidt

**Affiliations:** 1 School of Health and Society, Kristianstad University, Kristianstad, Sweden

**Keywords:** university student, stress, physical activity, eating behaviour, self-rated health, socio-demographics, lifestyle behaviour

## Abstract

**Background:**

Lifestyle behaviours are usually formed during youth or young adulthood which makes college students a particularly vulnerable group that easily can adopt unhealthy lifestyle behaviour.

**Aim:**

The aim of this cross-sectional study was to explore the influence of socio-demographic factors on Swedish university students’ lifestyle behaviours and self-rated health.

**Method:**

Data were collected from a convenience sample of 152 students using questionnaires consisting of a socio-demographic section followed by previously well-validated instruments. Data were analysed using descriptive statistics: *t*-tests, analysis of variance (ANOVA) and regression tests.

**Findings:**

The results of this study show that the lifestyle behaviours under study (physical activity, perceived stress and eating behaviours) as well as self-rated health can be predicted to a certain extent by socio-demographic factors such as gender, mother tongue and parents’ educational level. Male university students were shown to be physically more active than female students; the male students were less stressed and rated their overall health, fitness level and mental health higher. Female students were more prone to adopt unhealthy eating behaviours.

**Discussion:**

This study addresses gender differences and their influences on lifestyle behaviours; it provides both theoretical explanations for these differences as well as presents some practical implications of the findings.

## 1. Introduction

Previous studies clearly indicate that premature morbidity and mortality are primarily the result of unhealthy lifestyle behaviours (Boot, Rosiers, Meijman, & Van Hal, 2010; Dodd, Al-Nakeeb, Nevill, & Forshaw, 2010; Kvaavik, Batty, Ursin, Huxley, & Gale, 2010; World-Health-Organization, 2010). These studies focus to a large extent on lifestyle behaviours such as tobacco use (Boot et al., 2010; von Bothmer & Fridlund, 2005; World-Health-Organization, 2010), physical inactivity (Greene et al., 2011; Ottevaere et al., 2011; Ulla Diez & Perez-Fortis, 2010; von Bothmer & Fridlund, 2005; World-Health-Organization, 2010), unhealthy diet (Greene et al., 2011; Ottevaere et al., 2011; Ulla Diez & Perez-Fortis, 2010; von Bothmer & Fridlund, 2005; World-Health-Organization, 2010) and use of alcohol (Boot et al., 2010; von Bothmer & Fridlund, 2005; World-Health-Organization, 2010). Particular interest has been paid to the investigation of predictors of healthy lifestyles. At a younger age, genetic susceptibility, socio-demographic and behavioural factors are strongly related to mortality (Ford, Spallek, & Dobson, 2008). In other words, the association between socio-demographic factors and mortality is stronger at a younger age but weakens the older one gets (Mete, 2005). Among the socio-demographic factors gender has been shown to be one of the strongest socio-demographic factors that predict lifestyle behaviours (Ford et al., 2008; Johnson, 2005; Stock, Wille, & Kramer, 2001; von Bothmer & Fridlund, 2005).

## 2. Background

Lifestyle behaviours (defined as both causes and effects of one’s lifestyle) are usually formed during youth or young adulthood (Steptoe et al., 2002). The majority of university students are aged between 18 and 21 when entering university, a transition age to adulthood which is a time characterized by dramatic changes in life (Kwan, Cairney, Faulkner, & Pullenayegum, 2012). Students find themselves in a new, challenging and competitive environment; most of them are experiencing independence and responsibility for the first time in their lives. During university time they consequently adopt new health behaviours and there may be a risk that they continue with unhealthy lifestyle choices that were established during their university years, which makes them a risk group not only during these years but for the rest of their lives.

A substantial number of studies have been generated reporting that university students engage in unhealthy lifestyle behaviours such as substance abuse (Fromme, Corbin, & Kruse, 2008; Halperin, Smith, Heiligenstein, Brown, & Fleming, 2010; Vaughan, Corbin, & Fromme, 2009; H. R. White et al., 2006), physical inactivity (Greene et al., 2011; Ulla Diez & Perez-Fortis, 2010; Von Bothmer & Fridlund, 2005) and poor diet (Sira & Pawlak, 2010). In addition, many students experience stress caused by a number of factors, e.g. failing classes, competition or social pressure, which can result in other health problems such as insufficient sleeping patterns or a decrease in mental wellbeing.

Stress is known to influence health at various levels. It occurs when a person appraises a situation demand and/or challenge as exceeding available coping resources (D. von Ah D, 2004; Lazarus & Folkman, 1984). Researchers have shown that stress among university students can have effects on both academic performance and health (Campbell, Svenson, & Jarvis, 1992; D. von Ah D, 2004). University students are particularly exposed to stress due to the transitional nature of university life and the majority of students experience stress caused by academic commitments, financial pressures, and lack of time management skills (Misra, McKean, West, & Russo, 2000; Singh & Upadhyay, 2008). Moreover transition into university life is an important phase which might be particularly stressful due to the change in social network and the environment in general (Borsari, Murphy, & Barnett, 2007). This period also differs from the other periods of individuals’ lives since years at university often coincide with the transition from adolescence into adulthood, a period characterized by complex and often stressful processes influencing the formation of an adult (Hogan & Astone, 1986; Towbes & Cohen, 1996) Leisure satisfaction and fitness activities can act as stress buffers providing a sense of purpose and competence for university students (Misra et al., 2000; Ragheb & McKinney, 1993).

Eating habits of young students often change when they start studying. This can have different effects on both their health and personality. It is very common that university students gain weight in their first year at university. This gain can often be explained by the inconsistent eating habits which could be the result of stress, lifestyle, and changes in food and diet patterns (Levitsky, Halbmaier, & Mrdjenovic, 2004; Racette, Deusinger, Strube, Highstein, & Deusinger, 2005). Studies have also shown that university students suffer from eating disorders and skipping meals is a common habit among many. Eating habits affect the academic performance of students because study schedules or workload keep varying within and between semesters (Akdevelioglu & Gümüs, 2010; Thorsteinsdottir & Ulfarsdottir, 2008).

While the psychological stress, physical activity and various eating behaviours serve as important lifestyle and health indicators some researchers have suggested that the picture may be incomplete without an understanding of perceptions of one’s own health. Research has shown that self-rated health can provide relevant health information as well as information on lifestyle behaviours (Bopp, Braun, Gutzwiller, & Faeh, 2012). Therefore for the purpose of this paper, lifestyle behaviours are seen as a combination of self-rated health, psychological stress, physical activity and various eating behaviours.

To the author’s knowledge only few studies have attempted to link self-rated health, psychological stress, physical activity and various eating behaviours among university students with socio-demographic factors. One study by Dodd et al. clustering five lifestyle factors (smoking, physical activity, binge drinking, fruit and vegetable intake and psychological stress) in students in higher education in Great Britain found that nearly half of the sample (46%) was characterized as having an unhealthy lifestyle. This subgroup consisted of a larger percentage of female as well as a higher percentage of Asian or Asian British and Black or Black British students (Dodd et al., 2010). Another study, by Greene et al. (2011), revealed that of a sample consisting of 1689 university students enrolled at eight different universities in the United States 28.9% were overweight or obese and that only 5.5% met recommendations for fruit and vegetable intake. Other studies focus mainly on socio-demographic differences in health behaviours such as gender. Research conducted in Sweden shows that female students had healthier habits related to alcohol consumption (95% of women in the study had a low alcohol consumption pattern compared to 75% of men) and nutrition (21% of women reported healthy nutrition habits compared to 10% of men) but were more stressed (71% of women and 49% of men respectively), while male students showed a higher level of overweight (30% of men and 13% of women respectively) and were less interested in nutrition advice and health-enhancing activities (von Bothmer & Fridlund, 2005). One study conducted in Israel identified health behaviour differences by gender based on the social construction of masculinity and femininity (Soffer, 2010). This study indicates that, while women engage in ‘type 2 behaviours’- refraining from smoking and drinking, eating breakfast regularly and sleeping 7–8 hours per night- men engage in ‘type 1 behaviours’- physical exercise, refraining from snacking, and maintaining an appropriate body mass.

Sweden promotes itself as one of the most sporty nations worldwide (Swedish-Sports-Confederation, 2002) with strong emphasis on outdoor activities based on respect for nature and environment. Nearly half of the population aged 7–70 are members of sports clubs, most of which are non-profitable and run voluntarily. Especially among the young, physical activity takes a dominant position in their lives. Sweden being such a physically active nation (Martinez-Gonzalez et al., 2001) one would expect that people’s health consciousness in this respect would also have positive effects on people’s overall health, quality of life and wellbeing, also among the subpopulation of university students. A large number of articles have been published on health and lifestyle behaviours among university students in various countries possibly dominated by the United States and South-East Asia, but only a few studies focus on Swedish university students and their lifestyle behaviours. This paper’s purpose was therefore to explore how socio-demographic factors influence Swedish university students’ health-related behaviours.

## 3. Methods

### 3.1 Procedure

A cross-sectional study design was used in this paper which was carried out at Kristianstad University, which is a small university in the south of Sweden.

### 3.2 Participants

At the end of the spring term of 2011 full-time, on-campus university students were approached on two occasions, during two different lectures (microeconomics and research methods) offered to 2^nd^ and 3^rd^ year business students enrolled in the business administration programme in the section of Health and Society, Kristianstad University. There were 360 students enrolled in the programme at this time. The students were approached after class and asked by an assisting member of the business department to participate in the study. None of the students present in the classroom declined to participate and none of the questionnaires was returned blank. Altogether 152 students agreed to participate and completed the survey, which took approximately 5 minutes. The recruitment selection of the programme and classes was entirely based on convenience. Students were informed about the purpose of the study (to explore students’ lifestyle behaviour) and assured that their participation would be voluntary.

### 3.3 Data Collection

A survey was designed consisting of socio-demographic items and well-validated instruments from previous literature. The validated instruments which are discussed further in this section were translated from English into Swedish by a language expert, then another translator used the Swedish version to translate it back into English to ensure that there were no deviances from the original version. A third independent party validated both versions of the questionnaire by comparing the initial English version of the questionnaire and the version that was a result of the translation from Swedish to English, minor adjustments were made to insure the questionnaire’s consistency in both languages.

#### 3.3.1 Demographics

Socio-demographic data were collected using the first section of the designed questionnaire consisting of ten questions concerning gender, age, study course, current study term, body weight and height (self-reported by the students), mother tongue (native language) and marital status as well as mother’s and father’s education. Education of the parents has often been used as a proxy for social class (Chittleborough, Baum, Taylor, & Hiller, 2008; Telama, Laakso, Nupponen, Rimpela, & Pere, 2009). The sample consisted of 53.6% women and 46.4% men. The overall mean age was 23.4 (standard deviation (SD) = 3.6) years. Age, as well as study term, was grouped into few categories, so was mother tongue in order to compress data. Information about participants’ body mass index (BMI) was retrieved by using a formula dividing the individual’s body weight in kilograms by the square of their height. Body mass index is a universally used and recognized measure in medicine and other health-related disciplines that is used to categorize people’s weight into overweight, normal weight and underweight. An index <20 is classified as underweight, BMI 20-25 is rated normal or optimal weight and an index >25 is considered overweight. Participants’ mean BMI was 22.8 (SD 3.0). Divided by gender, the mean BMI was 24.0 (SD=2.8) for men, compared with 21.9 (SD=2.9) for women. Altogether 73.7% of the participants had normal weight, i.e. a BMI between 20-25.

#### 3.3.2 Psychological Stress

Stress was measured with Cohen’s ten-item Perceived Stress Scale (PSS) (Cohen, Kamarck, & Mermelstein, 1983), which is the most widely used psychological instrument for measuring perceived stress, which has also been used in recent studies using Swedish data (Bränström, Duncan, & Moskowitz, 2011). Items were designed to establish how unpredictable, uncontrollable and overloaded respondents find their lives. The questions ask about feelings and thoughts during the last month. Responses are made on a 5-point Likert scale (ranging from 0=never to 4=very often). Responses to questions 4, 5, 7 and 8 were then reversed in order to calculate the total score that could range from 0 (no stress) to 40 (very stressed). Cronbach’s alpha has been reported to be high ranging from 0.74 to 0.82 in a number of studies using the scale (Chaaya, Osman, Naassan, & Mahfoud, 2010; Dodd et al., 2010; Silverstein & Kritz-Silverstein, 2010; Sing & Wong, 2010). In the present study Cronbach’s alpha=0.842.

#### 3.3.3 Physical Activity

To measure self-reported physical activity the respondents were asked to complete Godin’s Leisure Time Exercise Questionnaire, a self-explanatory, brief, four-item questionnaire of habitual exercise habits (Godin & Shephard, 1985), that has been validated by a large number of studies (Bloss, Schork, & Topol, 2011; Scarmeas et al., 2009; Sebire, Standage, & Vansteenkiste, 2009; S. M. White, Wójcicki, & McAuley, 2009) including studies in Sweden (Jonsdottir, Rödjer, Hadzibajramovic, Börjesson, & Ahlborg Jr, 2010; Lagerros, Bellocco, Adami, & Nyren, 2009). To calculate the total physical activity score students were asked how often per week they performed strenuous, moderate and mild exercise for at least 15 minutes. Several examples of activities were provided to ensure that participants understood the concepts of strenuous, moderate and mild exercise. A formula then calculated the physical activity score taking the first three questions into account. The possible score range was from 0 to infinite. The fourth question (providing three answer alternatives) is concerned with frequency of activities and has not been taken into account for statistical measures since it did not fit the purpose of this paper.

#### 3.3.4 Self-Related Health

Since self-rated health has been reported to be a good predictor of health, wellbeing and quality of life (Bue Bjorner, 1996) it was included in this survey. In this study, self-rated health was defined as the individual’s perception and evaluation of their health. It was measured in a single-item question by asking the participants, “Overall, how would you rate your general health?” The question has been adapted from the Stanford Chronic Disease Self-Management Study (Lorig, 1996) and Bopp (Bopp et al., 2012). Since even mental health is included in most self-rated health questionnaires, and it has been shown that particularly university students are prone to stress due to peer pressure, financial issues, workload, etc and could consequently experience decreased mental wellbeing and/or unhealthy sleeping patterns, a one-item question regarding both self-rated mental health and self-rated sleeping quality was added, as well as a question concerning students’ self-rated fitness level.

#### 3.3.5 Eating Behaviours

One of the most widely used instruments in the research field of eating behaviours is the Three-Factor Eating Questionnaire (TFEQ) developed by Stunkard and Messick (Stunkard & Messick, 1985). Originally, this self-assessment questionnaire was designed to measure cognitive and behavioural components of eating in obese populations. It contains 51 items aggregated into three scales: cognitive restraint, disinhibition and hunger. In 2000, Karlsson et al. developed a revised version (TFEQ-R18) containing 18 items (Karlsson, Persson, Sjostrom, & Sullivan, 2000) covering the concepts of cognitive restraint, uncontrolled eating and emotional eating. Uncontrolled eating can also be understood as binge eating which is characterized by episodes of consuming abnormally large amounts of food. Cognitive restraint aims at limiting one’s food intake to prevent weight gain or to promote weight loss while emotional eating is the practice of consuming large quantities of food in response to feelings instead of hunger. In this study a shortened version of Karlsson et al.’s R18 was used. A factor analysis was performed resulting in three factors assigning three items to each eating behaviour. The items were chosen based on Angle et al.’s research paper from 2009 where the most loaded items for each of the three constructs were picked, factor loading range for uncontrolled eating was between 0.68 and 0.76, for cognitive restraint it was between 0.73 and 0.81, for emotional eating it was 0.81 and 0.87 (Angle et al., 2009). Cronbach’s alpha for this new version of the TFEQ, the TFEQ-R9 consisting thus of nine items in this paper is 0.765. Respondents’ answers could vary from strongly agree=1 to strongly disagree=4.

### 3.4 Statistical Analysis

A descriptive analysis was performed for all socio-demographic variables. A summary of socio-demographic information can be found in Table 1. Some of the socio-demographic variables were collapsed: thus, age was collapsed into three categories: ≤22 years, 23–27 years and ≥28 years; mother tongue was collapsed into two categories: Swedish, and other. To analyse the relationship between socio-demographic variables and lifestyle behaviours, *t*-tests were conducted for dichotomous variables and one-way analysis of variance (ANOVA) was performed for demographic dimensions with more than two categories. Multiple linear regression analysis was performed to analyse the combined effect of the socio-demographic variables (independent variables) on lifestyle behaviours (dependent variables). Since mother’s and father’s education contained more than two categories, dummy variables were created (0=uncompleted high school and high school diploma; 1=university degree). Age was included in the model as a continuous variable. To compute the regression models all of the variables were entered, and the non-significant variables were excluded from the model until it reached the point of best adjustment on R^2^ and significance of the variables included in the model which is also in line with some other studies with small sample size in the field (Ulla Diez & Perez-Fortis, 2010). The regression models were checked for multi-collinearity, and the tolerance values in the data vary between 0.798 and 0.913 which indicates that all models pass the test for multi-collinearity.

**Table 1:** Demographic characteristics of the sample (n=152)

Characteristics	Frequency (n)	Percentage (%)
Sex		
male	70	46.4
female	81	53.6
Age (yrs)		
≤22	79	52.0
22–27	53	34.9
≥28	20	13.1
Marital status		
single	68	45.6
in a relationship	81	54.4
Mother tongue		
Swedish	104	70. 7
other	43	29.3
Mother’s educational level		
uncompleted high school	28	18.5
high school diploma	56	37.1
university degree	67	44.4
Father’s educational level		
uncompleted high school	33	22.3
high school diploma	66	44.6
university degree	49	33.1

## 4. Results

The sample was evenly distributed concerning gender (53.6% women; 46.4% men), marital status (45.6% single; 54.4% in a relationship) and current study term (44.8% in terms 1–4; 55.2% in term 5 or higher). The majority of the university students were <22 years old (52%) and had Swedish as their mother tongue (70.7%). Most of their mothers and fathers had a high school diploma (37.1% and 44.6% respectively) or had studied at a university (44.4% and 33.1% respectively) (Table 1).

### 4.1 Socio-Demographics and Lifestyle Behaviours

In the bi-variate analysis physical activity was significantly related to students’ gender (p=0.077) and both the mother’s and the father’s educational level (p=0.096 and p=0.086 respectively) (Table 2).

**Table 2:** Comparison values for the socio-demographic characteristics in relation to the students’ lifestyle behaviours. Significant relationships are given in bold

Socio-demographics	Physical activity	Perceived stress	Self-rated overall health	Self-rated physical fitness level	Self-rated sleeping quality	Self-rated mental health
Gender						
male	52.64(32.130)	25.14 (6.791)	4.16 (0.927)	3.59 (1.097)	3.17 (1.142)	3.94 (1.006)
female	43.88(28.276)	29.79 (5.584)	3.78 (0.791)	3.27 (1.096)	3.37 (1.112)	3.72 (0.978)
p-value	0.077	0.000	0.007	0.081	0.281	0.163
Age (yrs)						
≤ 22	48.72 (29.103)	26.68 (6.383)	3.97 (0.905)	3.52 (1.108)	3.44 (1.206)	3.89 (0.974)
23–27	51.54	28.45 (6.146)	4.00 (0.734)	3.34 (1.108)	3.30 (0.952)	3.85 (0.928)
≥ 28	37.53 (25.463)	28.45 (8.432)	3.75 (1.070)	3.25 (1.070)	2.60 (0.995)	3.50 (1.192)
p-value	0.211	0.266	0.528	0.500	0.010	0.291
Mother tongue							
Swedish	50.37 (31.856)	27.48 (6.683)	4.02 (0.836)	3.40 (1.102)	3.38 (1.072)	3.82 (0.983)
other	43.55 (25.551)	27.60 (6.897)	3.72 (0.934)	3.40 (1.116)	2.93 (1.183)	3.77 (1.043)
p-value	0.214	0.926	0.059	0.966	0.028	0.784
Marital status							
single	48.14 (31.999)	26.67 (6.916)	3.96 (0.905)	3.41 (1.123)	3.28 (1.291)	3.90 (1.067)
in a relationship	48.26 (28.957)	28.27 (6.289)	3.96 (0.858)	3.42 (1.105)	3.30 (0.980)	3.78 (0.935)
p-value	0.981	0.147	0.961	0.965	0.928	0.468
Mother’s education							
9^th^ grade or less	37.71 (26.685)	28.74 (6.460)	3.64 (0.989)	3.04 (1.071)	3.07 (1.359)	3.57 (1.103)
high school	48.68 (29.048)	26.80 (6.878)	4.09 (0.745)	3.57 (0.988)	3.48 (1.009)	4.02 (0.904)
university	52.54 (32.454)	27.55 (6.510)	3.99 (0.896)	3.99 (0.896)	3.24 (1.074)	3.76 (1.001)
p-value	0.096	0.461	0.081	0.103	0.237	0.122
Father’s education							
9^th^ grade or less	38.59 (27.008)	27.78 (5.912)	4.00 (0.791)	3.24 (1.119)	3.39 (1.144)	3.88 (0.927)
high school	52.96 (33.356)	26.70 (6.616)	3.92 (0.900)	3.48 (1.099)	3.52 (1.070)	3.82 (0.975)
university	48.73 (27.718)	28.53 (6.871)	3.96 (0.912)	3.45 (1.138)	2.90 (1.065)	3.78 (1.085)
p-value	0.086	0.335	0.920	0.581	0.010	0.901

Socio-demographics	Eating behaviours					

	Uncontrolled eating		Cognitive restraint		Emotional eating	
Gender							
male	3.15 (0.748)		3.23 (0.833)		3.58 (0.487)	
female	3.13 (0.062)		3.01 (0.678)		3.05 (0.765)	
p-value	0.899		0.082		0.000	
Age (yrs)				
≤ 22	3.12 (0.692)		3.24 (0.637)		3.36 (0.605)	
23–27	3.22 (0.657)		3.02 (0.871)		3.21 (0.785)	
≥ 28	3.03 (0.708)		2.92 (0.844)		3.33 (0.817)	
p-value	0.527		0.116		0.471	
>Mother tongue			
Swedish	3.24 (0.644)		3.13 (0.763		3.33 (0.691)	
other	2.96 (0.667)		3.08 (0.759)		3.25 (0.742)	
p-value	0.019		0.703		0.540	
Marital status			
single	3.24 (0.663)		3.16 (0.701)		3.32 (0.727)	
in a relationship	3.05 (0.687)		3.07 (0.809)		3.27 (0.686)	
p-value	0.076		0.516		0.682	
Mother’s education			
9^th^grade or less	3.21 (0.740)		3.00 (0.822)		3.30 (0.893)	
high school	3.16 (0.713)		3.11 (0.773)		3.36 (0.652)	
university	3.09 (0.629)		3.17 (0.732)		3.25 (0.664)	
p-value	0.705		0.614		0.693	
Father’s education			
9^th^grade or less	3.05 (0.854)		3.19 (0.768)		3.23 (0.844)	
high school	3.30 (0.561)		3.15 (0.766)		3.36 (0.747)	
university	2.98 (0.668)		3.08 (0.750)		3.26 (0.541)	
p-value	0.030		0.803		0.608	

It is important to mention that a more liberal level of significance (level between 0.05 and 0.1) is being reported in this study since it can be seen as a validation/exploration study, aiming at observing patterns in the Swedish context, and comparing it to the similar studies in the field, yet performed in different settings. Higher physical activity was reported by male students. The higher the mother’s education the higher the physical activity score, though with regard to the father’s educational level the highest physical activity scores were observed when students’ fathers held a high school diploma. The lowest physical activity scores were observed when parents’ education fell in the category 9th grade or less. In the multiple regression analyses only gender was generated as a significant variable in the adjusted model, explaining 2.8% of the variance (R=0.167; R^2^=0.028; p=0.048) (Table 3).

**Table 3:** Summary of the multiple regression analysis of the effect of socio-demographic variables on lifestyle behaviours

	β	*t*	p	CI^B^
Predicted physical activity (R=0.167; R^2^=0.028; p=0.048)					
gender	0.167	1.993	0.048	0.082–20.093
Predicted perceived stress (R=0.382; R^2^=0.146; p=0.000)					
gender	0.382	4.803	0.000	-7.161– -2.984
Predicted overall health (R=0.310; R^2^=0.096; p=0.001)					
gender	0.256	3.148	0.002	0.168–0.737
mother tongue	0.188	2.310	0.022	0.053–0.690
Predicted sleeping quality (R=0.363; R^2^=0.132; p=0.000)					
age	-0.166	-2.045	0.043	-0.098– -0.002
mother tongue	0.191	2.355	0.020	0.076–0.876
father’s education (dummy)	-0.263	-3.281	0.001	-1.001– -0.248
Predicted uncontrolled eating (R=0.263; R^2^=0.069; p=0.008)					
mother tongue	0.193	2.328	0.021	0.043–0.530
father’s education (dummy)	-0.167	-2.019	0.045	-0.469– -0.005
Predicted cognitive restraint (R=0.211; R^2^=0.044; p=0.046)					
gender	0.157	1.869	0.064	-0.014–0.487
age	-0.139	-1.660	0.099	-0.062–0.005
Predicted emotional eating (R=0.360; R^2^=0.130; p=0.000)					
gender	0.360	4.520	0.000	0.289–0.739

In the bi-variate analysis with PSS as a dependent variable, significant differences were only found with students’ gender (p=0.000) (Table 2). Higher perceived stress was reported among the women. Multiple regression yielded the same results; only this variable was included in the final equation, and it accounted for 14.6% of the total variance (R=0.382; R^2^=0.146; p=0.000) (Table 3).

In the bi-variate analysis self-rated overall health was significantly related to students’ gender (p=0.007), mother tongue (p=0.059) and the mother’s education (p=0.081) (Table 2). Higher rating on overall health was reported among male students and among students with Swedish as their mother tongue. Students with mothers who held a high school diploma reported higher rating on overall health. The multiple regression model included gender and mother tongue as variables accounting for nearly 10% of variance (R=0.310; R^2^=0.096; p=0.001) (Table 3).

In the bi-variate analysis with self-rated physical fitness as a dependent variable significant differences were only found with gender (p=0.081) (Table 2). Male students rated themselves physically fitter than female students did. Multiple regression analyses did not generate a model for self-rated physical fitness as a variable.

In the bi-variate analysis self-rated sleeping quality as a dependent variable was significantly related to age (p=0.010), mother tongue (p=0.028) and the father’s education (p=0.010) (Table 2). The older the participants the worse they rated their sleeping quality. Sleeping quality was rated significantly lower by students with a foreign background. Students whose fathers held a high school diploma rated the sleeping quality higher compared with students whose fathers had either a lower education or had a university degree. The multiple regression analyses yielded the same results; the model included the same three variables as predictive variables and explained 13.2% of the total variance (R=0.363; R^2^=0.132; p=0.000) (Table 3).

With regard to self-rated mental health as a dependent variable no socio-demographic dimension was significantly related to this variable in either the bi-variate or the multiple regression analysis.

In the bi-variate analysis uncontrolled eating behaviour was significantly related to mother tongue (p=0.019), marital status (p=0.076) and the father’s education (p=0.030) (Table 2). Uncontrolled eating behaviours were significantly higher among participants who lived in a relationship. Students with a foreign background also showed a higher tendency for uncontrolled eating as did students whose fathers had a high school diploma. The multiple regression model included mother tongue and father’s educational level as variables, accounting for 6.9% of variance (R=0.263; R^2^=0.069; p=0.008) (Table 3).

In the bi-variate analysis with cognitive restraint as a dependent variable significant differences were only found with gender (p=0.082) (Table 2). In female students a higher tendency towards cognitive restraint was reported. Besides gender the multiple regression model included age as variable, accounting for nearly 4.4% of variance (R=0.211; R^2^=0.044; p=0.046) (Table 3).

In the bi-variate analysis with emotional eating as a dependent variable significant differences were only found with gender (p=0.000) (Table 2). Emotional eating tends to be higher among women. Multiple regression yielded the same results; only gender was included in the final equation, and it accounted for 13% of the total variance (R=0.360; R^2^=0.130; p=0.000) (Table 3).

## 5. Discussion

The study’s results give insight into how socio-demographic factors – gender, age, mother tongue, marital status and the mother’s and father’s educational level – influence Swedish university students’ lifestyle behaviours. Overall, the findings show that gender and mother tongue seem to be the strongest predictors of certain behaviours. Interestingly, even the educational level of the parents was shown to be related to the students’ lifestyle behaviours, albeit on the lower significance level of 10%. These findings are in agreement with a previous study by Ford and colleagues (Ford et al., 2008). They found that many socio-demographic factors have their origins in early life; parental socio-economic status influences a person’s educational and occupational aspirations and achievements, which in turn affect their own socio-economic status. These factors influence the establishment of lifestyle characteristics such as tobacco and alcohol consumption, levels of physical activity, weight maintenance and other health-related behaviours (Ford et al., 2008).

Overall, male university students in this study were physically more active and less stressed compared with female students; male participants also rated their overall health, fitness level and mental health higher than the female participants did.

When it comes to the differences in physical activity between male and female students one explanation could be that men and women have different motivations for being physically active (Maltby & Day, 2001). While men usually are motivated to exercise by intrinsic factors such as challenge and enjoyment, women tend to be motivated by extrinsic motivation factors such as appearance improvement, weight management and ill-health avoidance (Egli, Bland, Melton, & Czech, 2011; Kilpatrick, Hebert, & Bartholomew, 2005). Egli et al. (2011) have found that intrinsic motivation, which is more common in men, is usually reflected in a higher level of physical activity while extrinsic motivation, which is more common in women, is usually reflected in a decreasing level of physical activity. Thus the differences in motivation factors associated with physical activity could also explain the differences in self-rated fitness between sexes in this study.

The results concerning differences between men and women in reported level of stress could be explained by findings of Misra and McKean who found that women react to certain stressors such as frustration, self-imposed stress and pressure in relation to academia with a higher level of stress than men (Misra & McKean, 2000). Another study found that women not only have a higher overall level of stress but also react with a higher level of stress to problems in familial relationships, social relationships and daily hassles (Brougham, Zail, Mendoza, & Miller, 2009). Moreover, the higher stress level among female students might be explained by the fact that females are more prone to report stress than males (Weinstein & Laverghetta, 2009). Though the female participants were more stressed than the male participants, they rated their sleeping quality better than men though the number of hours slept did not significantly differ. Previous research findings suggest that male students were involved in substance abuse (e.g. alcohol) considerably more than female students (Lawrence, Abel, & Hall, 2010) which could potentially explain gender difference in quality of sleep (Buboltz Jr et al., 2009) as shown in the study.

Gender differences in perceptions of one’s own mental health found in this paper are in line with previous findings (H. Li & Prevatt, 2007; Sutton & Farrall, 2005). One reason for this difference might lie in the female’s tendency to report more severe mental health problems than male students (H. Li & Prevatt, 2007). Alternatively, females might experience a wider range of life events as stressful compared to men who react to a more limited range of stressful events (Huijun Li et al., 2008). Similarly the differences in the rating of health between men and women could be attributed to the gender-embedded reporting differences where female students are more inclined towards a more negative view of their health then male students.

Concerning eating behaviours, women scored significantly higher regarding cognitive restraint (albeit on the lower significance level) and emotional eating, indicating that they might be more conscious about their weight and more prone to emotional imbalance compared with men (Mintz & Kashubeck, 1999). Rather than using physical activity as a means to control weight women are more likely to develop an unhealthy relationship with food and adopt unhealthy eating behaviours (Mintz & Kashubeck, 1999). Significant differences concerning uncontrolled eating were found in marital status (weakly significant), mother tongue and father’s education. This kind of eating behaviour was detected mainly among students with a foreign background, students in relationships and students with fathers that reached a university degree. Even though these differences were found, only mother tongue and father’s education could explain the variation in uncontrolled eating behaviour.

## 6. Conclusions

The findings of this study reveal that there is a great need to address health and lifestyle of university students since their lifestyle behaviours may have an impact on their future lives and wellbeing. For this reason, health promotion programmes at institutions of higher education may be beneficial in raising student awareness of their present and future health in relation to their lifestyle behaviours. Several such programmes have already been implemented at a number of universities in Sweden. These programmes include a lifestyle check that gives students the possibility to fill out questionnaires similar to the questionnaire used in this study and get immediate response and further information and guidance depending on the test results. Continuous promotion of a healthy lifestyle by students to students could be yet another way forward since peer promotion can be a powerful tool in influencing healthy lifestyle behaviour. Initiation of specific health promoting events independently or as a part of sport and other club activities could be yet another way of encouraging healthy lifestyle behaviour.

The study is, however, not without limitations. Sample size is one of the major limitations of this study. The sample consisted of students majoring in business administration, studying at a small university in the south of Sweden. The results of the study may not necessarily apply to all university students at Swedish universities and possibly if investigated at larger universities the results would have been slightly different. Thus investigating student lifestyle behaviours taking into account their field of study or type of university attended could potentially be an interesting topic for future research. Comparative studies between different countries could be yet another interesting area for future research, where students’ national rather than ethnic differences could be investigated in relationship to lifestyle behaviours.

This study used a questionnaire as a means of data collection. While there are many positive aspects of this tool, it also contains inherent limitations associated with the use of quantitative data collection techniques, where respondents could answer certain questions without even reading it. Moreover self-reporting technique is associated with the inaccuracy of the information provided; however examination of the outliers (as performed in this study) could possibly illuminate some biases associated with the technique. Future studies could adopt a broader view on behavioural lifestyles through triangulation, where qualitative methods e.g. focus groups and observations could lead to collection of richer data. Yet another and potentially interesting future research project could investigate how lifestyle behaviours of students and particularly business students change over time, by collecting data from young business professionals, which could be especially interesting taking into consideration that many firms today set healthy lifestyle as one of their strategic goals.
